# Supplementary material for: Assessing the impact of global warming on the distributions of Allium stipitatum and Kelussia odoratissima in the Central Zagros using a MaxEnt model
Source: PLoS One. 2025 Apr 16;20(4):e0321167. doi: 10.1371/journal.pone.0321167 (PMC12002440; doi:10.1371/journal.pone.0321167)
Supplement: Table S1 — The variance inflation factors (VIFs) of the remained variables of Allium stipitatum and Kelussia odoratissima. (DOCX) [file pone.0321167.s001.docx]

**Table S1** The variance inflation factors (VIFs) of the remained variables of Allium *stipitatum* and *Kelussia odoratissima*

| VIFs of the remained variables of *Allium stipitatum* | |
| --- | --- |
| Variables | VIF |
| Altitude | 8.605712 |
| Aspect | 1.235876 |
| BIO13 | 5.212245 |
| BIO14 | 2.517674 |
| BIO15 | 6.831514 |
| BIO17 | 3.263751 |
| BIO18 | 5.704798 |
| BIO2 | 2.919034 |
| BIO3 | 7.904629 |
| BIO7 | 3.758591 |
| BIO8 | 5.49161 |
| OC | 5.866811 |
| Clay | 2.515379 |
| EC | 1.553741 |
| pH | 7.293012 |
| Sand | 6.595414 |
| Slope | 1.072506 |
| Silt | 1.765481 |

**Table S2** The variance inflation factors (VIFs) of the remained variables of *Kelussia odoratissima*

| **VIFs of the remained variables of *Kelussia odoratissima*** | |
| --- | --- |
| **Variables** | **VIF** |
| Altitude | 9.325715 |
| Aspect | 1.166164 |
| BIO13 | 5.277888 |
| BIO15 | 6.449375 |
| BIO16 | 5.645143 |
| BIO17 | 3.763456 |
| BIO18 | 2.100771 |
| BIO2 | 3.496793 |
| BIO4 | 8.11387 |
| OC | 5.577801 |
| pH | 6.784426 |
| Sand | 1.588783 |
| Slope | 1.120368 |
