# Supplementary material for: Assessing the impact of global warming on the distributions of Allium stipitatum and Kelussia odoratissima in the Central Zagros using a MaxEnt model
Source: PLoS One. 2025 Apr 16;20(4):e0321167. doi: 10.1371/journal.pone.0321167 (PMC12002440; doi:10.1371/journal.pone.0321167)
Supplement: Attachment — Submitted filename: Reviewer Comment-PONE-D-24-55210.docx [file pone.0321167.s002.docx]

Manuscript Number: PONE-D-24-55210

Title-Two plant species, *Allium stipitatum* and *Kelussia odoratissima*, will suffer due to

climate change in Central Zagros

Comments-.

The manuscript is well-written and effectively elucidates the impact of climate change on two significant plant species in Iran. I have some minor queries:

1. **Selection of Plant Species**: Why were only these particular plants chosen for the study? Under similar conditions, are other plants more resistant to climate change?
2. **Vulnerability Comparison**: Why are these plants more susceptible to climate change compared to other species in the area? Do the climate factors discussed in this study have a lesser impact on other regional plants?
3. **Historical Climate Data**: Is there data indicating changes in climate factors such as temperature, rainfall, and soil conditions over past decades?
4. **Future Climate Projections**: What are the anticipated changes in climate conditions in the future compared to the present scenario?
5. **Mitigation Strategies**: Considering that climate change is a global phenomenon, what potential solutions could be implemented in these areas to mitigate its effects?
